# Supplementary material for: Diagnosis of brucellosis: Combining tests to improve performance
Source: PLoS Negl Trop Dis. 2024 Sep 5;18(9):e0012442. doi: 10.1371/journal.pntd.0012442 (PMC11407618; doi:10.1371/journal.pntd.0012442)
Supplement: Attachment — Submitted filename: CoverLetter27052024.docx [file pntd.0012442.s002.docx]

Dear Editor,

Please find enclosed the revised version of our manuscript entitled ‘Diagnosis of brucellosis: combining tests to improve performance’ by Paul Loubet, Chloé Magnan et al.

We would like to express our gratitude to the reviewer for the valuable feedback. Below, we have addressed each of the comments in detail.

We appreciate your attention to this matter and hope that our responses adequately address the reviewer' comments. We look forward to hearing from you soon.

Yours faithfully,

Professor Jean-Philippe LAVIGNE

**Reviewer 1’s comments:**

Comments to the Author

-A minor modification is needed. Please see the enclosed revised manuscript.

The authors appreciate the comments provided by the reviewer. These comments have been addressed point by point in the revised version of the manuscript. We have made the necessary modifications as per the reviewer’s suggestion.

The authors appreciate the valuable comments from the reviewer and all of the questions have been addressed in the revised version of the manuscript.

Abstract: P. 2

-Ln 4: We have corrected the typo as suggested

-Ln 12: We have corrected the typo as suggested

Introduction: P. 4

-P. 4 Ln 16: “disease-free” We modified accordingly in the revised version of the manuscript

-P.5 Ln 18: We have corrected the typo as suggested

Methods:

-P. 7 Ln 4: We have made the necessary modification as per your suggestion

-P. 7 Ln 6: “an acidic medium” We modified accordingly in the revised version of the manuscript

-P. 7 Ln 12: We have corrected the typo as suggested

-P. 8 Ln 1: We have made the necessary modification as per your suggestion

-P. 8 Ln 4: The sentence concerned the different ELISA techniques and not juts one ELISA. We adjusted the sentence accordingly

-P. 8 Ln 7: We modified accordingly in the revised version of the manuscript

-P. 8 L, 13: We have corrected the typo as suggested

-P. 8 Ln 15: We modified accordingly in the revised version of the manuscript

Results:

P. 9 Ln 20: “3439 were negative” We modified accordingly in the revised version of the manuscript

P. 10 Ln 20:”best diagnostic algorithm for diagnosing brucellosis”. We modified accordingly in the revised version of the manuscript

P. 10 Ln 24: “dilutions”. We modified accordingly in the revised version of the manuscript

P. 11 Ln 17: “dilutions”. We modified accordingly in the revised version of the manuscript

P. 11 Ln 25: “false positives”. We modified accordingly in the revised version of the manuscript

P. 12 Ln 22: “false positives”. We modified accordingly in the revised version of the manuscript

Discussion

P. 13 Ln 15: We have made the necessary modification as per your suggestion

P. 13 Ln 18: We have made the necessary modification as per your suggestion

P. 14 Ln 4: We have corrected the typo as suggested

P. 14 Ln 13: We have made the necessary modification as per your suggestion

P. 14 Ln 25: We have corrected the typo as suggested

P. 15 Ln 7: We have made the necessary modification as per your suggestion

Tables

Table 2: We have made the necessary modification as per your suggestion

-The manuscript didn't show any novel test. However, the proposed test may help to improve the serologic test to improve the diagnosis of brucellosis.

We thank the reviewer for their positive comment. We agree with this reviewer, the techniques are not new. In fact, that’s the principle of our paper: to use old techniques, those that are currently the most widespread in the world, and add their performance to obtain a much more effective diagnostic tool for diagnosing brucellosis (compared to each test taken separately).

**Editorial office**

1) Thank you for stating" The local ethics committee (Institutional Review Board of Nîmes University Hospital; registration number n°2024.03.05) approved their use in this research. Before inclusion, patients were informed of the study and their rights to oppose the use of their serology results.

Please include a statement that formal consent was obtained (must state whether verbal/written) OR the reason consent was not obtained (e.g. anonymity). NOTE: If child participants, the statement must declare that formal consent was obtained from the parent/guardian.]

A verbal consent was obtained from the different patients. As each tube came from different centers in France, each biologist had to tell us whether or not the tube could be included in a serum bank for future analyses and if the patient accepted these analyses.

2) We note that you have included "Author Summary" in the Manuscript file. Please note that this should be [150 to 200 words] long. The aim should be to make your findings accessible to a wide audience that includes both scientists and non-scientists. Sample summaries can be found on our website under Submission Guidelines:
[https://journals.plos.org/plosntds/s/submission-guidelines#loc-parts-of-a-submission](https://journals.plos.org/plosntds/s/submission-guidelines#loc-parts-of-a-submission)

We modified or “Author Summary” in the new version of our manuscript.

3) Please ensure that the CRediT author contributions listed for every co-author are completed accurately and in full.

At this stage, the following Authors/Authors require contributions: Paul Loubet, Chloé Magnan, Florian Salipante, Théo Pastre, Anne Keriel, David O'Callaghan, Albert Sotto, and Jean-Philippe Lavigne. Please ensure that the full contributions of each author are acknowledged in the "Add/Edit/Remove Authors" section of our submission form.

The list of CRediT author contributions may be found here: <https://journals.plos.org/plosntds/s/authorship#loc-author-contributions>

We modified accordingly.

4) We do not publish any copyright or trademark symbols that usually accompany proprietary names, eg ©,  ®, or TM  (e.g. next to drug or reagent names). Therefore please remove all instances of trademark/copyright symbols throughout the text, including:® on pages: Brucellacapt® on page no 2, 7, WRIGHT®, on page no 7, Brucellacapt® on page no 7, 8, 9, 11, 13, 14, 22, and 24.

We modified accordingly.

5) Please upload a copy of  figure1 which you refer to in your text on the manuscript file. Or, if the figure is no longer to be included as part of the submission please remove all reference to it within the text.

We added the file.

6) Thank you for stating"Data are available in Nîmes University Hospital intranet file :///Users/jphlavigne/Downloads/RE%20_Article_se%CC%81ro_Brucella/20240329_RAPPORTSTAT_Brucella_Rose_Bengale.html#pr%C3%A9dictions-avec-rose-bengale-sat

This link reaches a error page. Please amend this to a new working link

The link is currently inaccessible due to the intranet firewall. The data supporting the findings of this study can be available from the corresponding author upon reasonable request.
