# Supplementary material for: Diagnosis of brucellosis: Combining tests to improve performance
Source: PLoS Negl Trop Dis. 2024 Sep 5;18(9):e0012442. doi: 10.1371/journal.pntd.0012442 (PMC11407618; doi:10.1371/journal.pntd.0012442)
Supplement: Attachment — Submitted filename: CoverLetter31072024.docx [file pntd.0012442.s003.docx]

Dear Editor,

Please find enclosed the revised version of our manuscript entitled ‘Diagnosis of brucellosis: combining tests to improve performance’ by Paul Loubet, Chloé Magnan et al.

We would like to express our gratitude to the reviewer for the valuable feedback. Below, we have addressed each of the comments in detail.

We appreciate your attention to this matter and hope that our responses adequately address the reviewer' comments. We look forward to hearing from you soon.

Yours faithfully,

Professor Jean-Philippe LAVIGNE

Response to Reviewers (May 27^th^, 2024)

**Reviewer 1’s comments:**

Comments to the Author

-A minor modification is needed. Please see the enclosed revised manuscript.

The authors appreciate the comments provided by the reviewer. These comments have been addressed point by point in the revised version of the manuscript. We have made the necessary modifications as per the reviewer’s suggestion.

The authors appreciate the valuable comments from the reviewer and all of the questions have been addressed in the revised version of the manuscript.

Abstract: P. 2

-Ln 4: We have corrected the typo as suggested

-Ln 12: We have corrected the typo as suggested

Introduction: P. 4

-P. 4 Ln 16: “disease-free” We modified accordingly in the revised version of the manuscript

-P.5 Ln 18: We have corrected the typo as suggested

Methods:

-P. 7 Ln 4: We have made the necessary modification as per your suggestion

-P. 7 Ln 6: “an acidic medium” We modified accordingly in the revised version of the manuscript

-P. 7 Ln 12: We have corrected the typo as suggested

-P. 8 Ln 1: We have made the necessary modification as per your suggestion

-P. 8 Ln 4: The sentence concerned the different ELISA techniques and not juts one ELISA. We adjusted the sentence accordingly

-P. 8 Ln 7: We modified accordingly in the revised version of the manuscript

-P. 8 L, 13: We have corrected the typo as suggested

-P. 8 Ln 15: We modified accordingly in the revised version of the manuscript

Results:

P. 9 Ln 20: “3439 were negative” We modified accordingly in the revised version of the manuscript

P. 10 Ln 20:”best diagnostic algorithm for diagnosing brucellosis”. We modified accordingly in the revised version of the manuscript

P. 10 Ln 24: “dilutions”. We modified accordingly in the revised version of the manuscript

P. 11 Ln 17: “dilutions”. We modified accordingly in the revised version of the manuscript

P. 11 Ln 25: “false positives”. We modified accordingly in the revised version of the manuscript

P. 12 Ln 22: “false positives”. We modified accordingly in the revised version of the manuscript

Discussion

P. 13 Ln 15: We have made the necessary modification as per your suggestion

P. 13 Ln 18: We have made the necessary modification as per your suggestion

P. 14 Ln 4: We have corrected the typo as suggested

P. 14 Ln 13: We have made the necessary modification as per your suggestion

P. 14 Ln 25: We have corrected the typo as suggested

P. 15 Ln 7: We have made the necessary modification as per your suggestion

Tables

Table 2: We have made the necessary modification as per your suggestion

-The manuscript didn't show any novel test. However, the proposed test may help to improve the serologic test to improve the diagnosis of brucellosis.

We thank the reviewer for their positive comment. We agree with this reviewer, the techniques are not new. In fact, that’s the principle of our paper: to use old techniques, those that are currently the most widespread in the world, and add their performance to obtain a much more effective diagnostic tool for diagnosing brucellosis (compared to each test taken separately).

Response to Reviewer/Editor (June 3^rd^, 2024)

Editor comments:

-This is primarily a review and this topic has been very much addressed already:

We respectfully disagree with this assessment. Our paper is not a review, but rather it presents original research. While the topic of brucellosis has indeed been studied, our research provides a unique perspective and contributes new insights that have not been previously explored in depth.

- This ms does not advance the field:

We understand your concern. However, our study does not aim to introduce a novel test. Instead, we propose a new approach to improve the serological test for diagnosing brucellosis. This approach, which utilizes routine diagnostic tests of brucellosis in an algorithm, has not been addressed in previous works. We believe that our research can significantly contribute to the field by enhancing the accuracy and efficiency of brucellosis diagnosis.
